# Epidemiological and spatial–temporal clustering characteristics of pertussis in Southwest China, 2013–2024

**DOI:** 10.3389/fpubh.2025.1620429

**Published:** 2026-01-12

**Authors:** Ju Wang, Jiawei Xu, Dayong Xiao, Baisong Li, Yu Xiong, Jule Yang, Wenshuang Wei, Longyu Chen, Qi Wang, Yao Qin, Yang Liu, Han Zhao, Jiang Long, Li Qi

**Affiliations:** 1 Department of Infectious Disease Prevention and Control, Chongqing Municipal Center for Disease Control and Prevention (Chongqing Academy of Preventive Medicine), Chongqing, China; 2 Department of Infectious Disease Prevention and Control, Chongqing Municipal Key Laboratory of Highly Pathogenic Microorganisms, Chongqing, China; 3 Department of Basic Medical Education, Dazhou Vocational College of Traditional Chinese Medicine, Dazhou, Sichuan, China; 4 School of Public Health, Southwest Medical University, Luzhou, Sichuan, China; 5 Department of Infectious Disease Prevention and Control, Sichuan Center for Disease Control and Prevention, Chengdu, Sichuan, China

**Keywords:** pertussis, resurgence, spatiotemporal analysis, vaccine, whooping cough

## Abstract

**Background:**

Despite high childhood vaccination coverage, pertussis has resurged in China in recent years. Understanding its evolving epidemiology is critical for guiding public health interventions. This study analyzed spatiotemporal trends and identified key drivers of pertussis notification rates in Chongqing, China (2013–2024).

**Methods:**

We analyzed longitudinal surveillance data of pertussis cases from the National Notifiable Infectious Disease Reporting System (NNDRS) in Chongqing, China (2013–2024). Demographic and vaccination coverage data were obtained from the Basic Information System (a subsystem of the NNDRS) and the National Immunization Program Information System (NIPIS), respectively. We employed joinpoint regression to quantify long-term notification rate trends, interrupted time-series analysis to evaluate the impact of national diagnostic criteria revisions (2017, 2024), spatiotemporal scan statistics to identify high-risk space–time clusters, and multilevel linear mixed-effects models to determine district-level factors associated with notification rates, while accounting for random variations across districts and years.

**Results:**

From 2013 to 2024, 33,226 pertussis cases were notified in Chongqing. A dramatic surge occurred in 2024, accounting for 68.55% (*n* = 22,777) of all cases. A distinct epidemiologic shift was observed in age distribution: During 2013–2023, infants < 1 year had the highest notification rate and were the largest age group (56.8%, 5,939/10,449), with 44.7% (2,658/5,939) being aged < 3 months. However, in 2024, although infants (<1 year) retained the highest notification rate, children aged 5–10 years constituted the highest group by case count (*n* = 13,868), a case count 7.4 times greater than that in infants (*n* = 1,873). Joinpoint regression showed a statistically significant upward trend in the annual pertussis notification rate (average annual percent change [*AAPC*] = 32.16%; 95% confidence interval [*CI*]: 5.40–65.72). The most pronounced increase was observed among children aged 5–10 years (AAPC = 94.91%; 95% CI: 55.18–144.81). The 2024 revision of national pertussis diagnostic criteria was associated with an immediate 26.5-fold increase in pertussis notifications (Incidence Rate Ratio [*IRR*] = 26.46; 95% *CI*: 17.52–39.95; *p* < 0.001). Multilevel regression identified higher district-level income levels (*β* = 0.56, *p* < 0.001) and the 2024 diagnostic criteria revision (*β* = 5.52, *p =* 0.013) as significant determinants of higher pertussis notification rates. No significant association was found with district-level vaccination coverage.

**Conclusion:**

This study documents a substantial resurgence of pertussis in Chongqing from 2013 to 2024, characterized by a notable change in age distribution. Although infants (aged <1 year) maintained the highest notification rate, school-aged children (5–10 years) emerged as the largest case group in 2024. Consequently, establishing seroepidemiological surveillance to directly quantify population immunity is essential for designing targeted and effective control measures.

## Introduction

Pertussis, an acute respiratory infection caused by *Bordetella pertussis* (*B. pertussis*) (1), has afflicted humans for centuries (2). This highly contagious disease impacts individuals across all age groups and can be fatal in unvaccinated populations, particularly in young infants (3).

Recent reports indicate a “resurgence” of pertussis (4–6), accompanied by substantial outbreaks globally (6–8). Pertussis has reemerged as a major public health threat, particularly for infants and young children (3, 9). Despite sustained high coverage (>99%) of the three-dose diphtheria-tetanus-acellular pertussis (DTaP) vaccine series in China over the past decade, the pertussis notification rate has exhibited a fluctuating but overall increasing trend (6). Pertussis remains a persistent public health challenge (6). China is one of the countries with the highest incidence of pertussis in recent years (1, 6).

Chongqing, a municipality in southwestern China, has experienced a dramatic 14.59-fold increase in its pertussis notification rate, from 0.25 per 100,000 in 2004 to 3.66 per 100,000 in 2018 (10). This rate exceeds the national average and imposes a substantial medical economic burden (10, 11).

Understanding pertussis transmission patterns, particularly spatial and temporal dynamics, is crucial for effective control. However, such analyses have been conducted in various regions (12–16) and in other Chinese provinces (17, 18). These patterns are highly region-specific, influenced by local demographic, environmental, and surveillance factors. Therefore, despite the valuable insights from previous studies, there remains a critical need for localized analyses to inform targeted public health interventions in specific high-burden areas. This study aimed to investigate the shifting epidemiology of pertussis in Chongqing from 2013 to 2024 by identifying priority populations, analyzing long-term trends, and detecting high-risk spatiotemporal clusters, thereby providing an evidence-based foundation for optimizing local control strategies.

## Methods

### Study area

**Figure 1:**
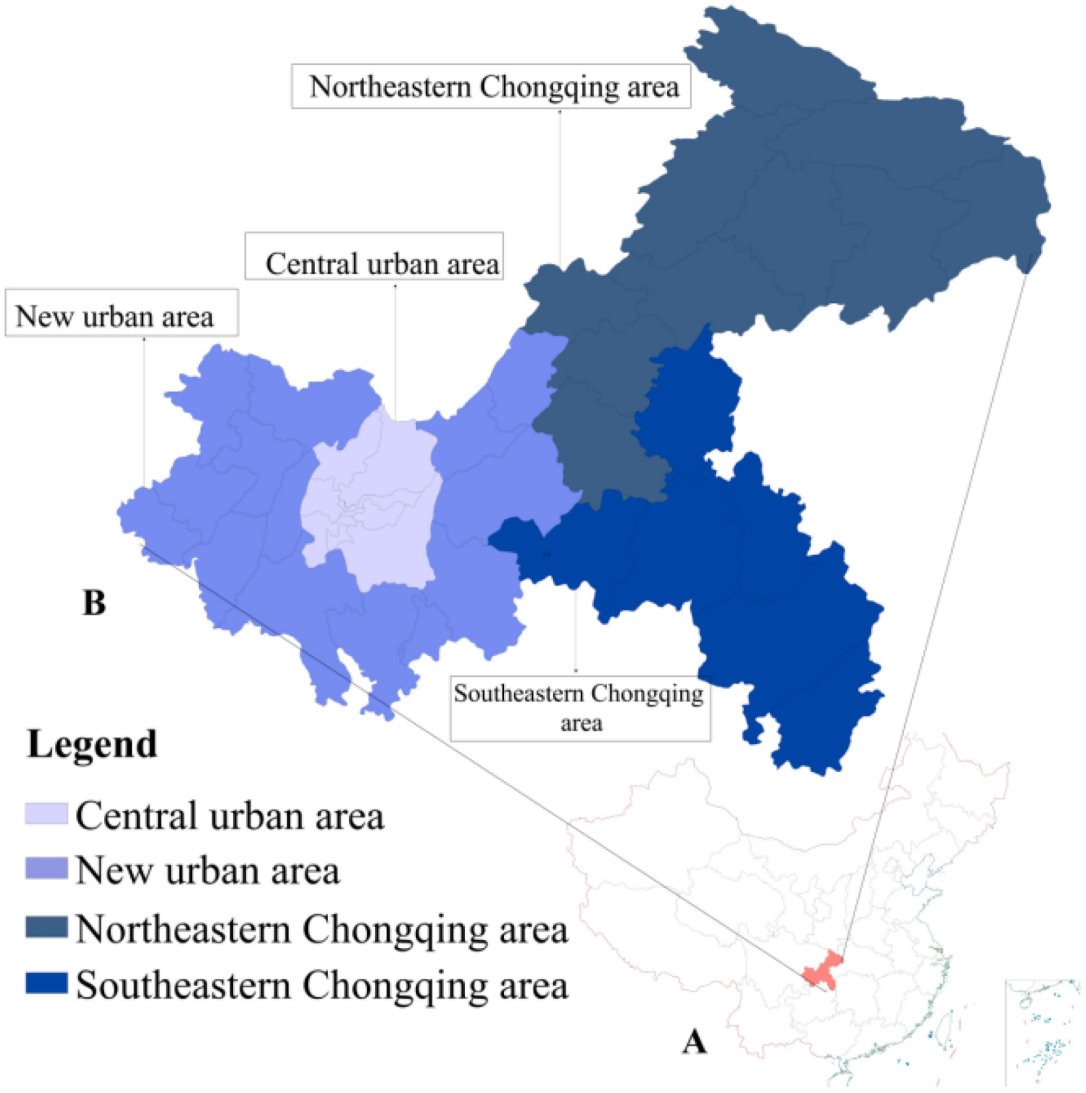
Geographical division of Chongqing. **(A)** Location of Chongqing within China. **(B)** Four geographical regions and their constituent administrative divisions in Chongqing.

Chongqing, one of China’s four direct-administered municipalities, has a population exceeding 34 million. It is located in southwestern China (228°10′–32°13′N, 105°11′–110°11′E) and encompasses an area of 82,400 km^2^. Chongqing has a humid subtropical monsoon climate characterized by hot summers, mild winters, and a rainy season that coincides with the hottest period. This results in a long frost-free period, abundant rainfall, and frequent humid and cloudy days (19). According to the Chongqing Municipal People’s Government (20), the city is segmented into four regions: the Central urban area (9 districts), the New urban area (12 districts/counties), the Southeastern Chongqing area (6 districts/counties), and the Northeastern Chongqing area (11 districts/counties), as illustrated in Figure 1.

### Data collection

Demographic and epidemiological data for reported pertussis cases from 2013 to 2024, including sex, age, date of symptom onset, and residential address, were extracted from the National Notifiable Infectious Disease Reporting System (NNDRS). Annual population denominators for each county and district were obtained from the Basic Information System, a subsystem of the NNDRS.

Vaccination coverage data for pertussis-containing vaccines were sourced from the National Immunization Program Information System (NIPIS). In China, acellular pertussis vaccines (aPVs) were introduced in 2007 and had completely replaced whole-cell pertussis vaccines (wPVs) nationwide by 2013, the starting year of our study (21). Throughout the study period (2013–2024), the national childhood immunization schedule consistently mandated a three-dose primary series of diphtheria-tetanus-acellular pertussis (DTaP) vaccine at 3, 4, and 5 months of age, followed by a booster dose at 18 months (22). Vaccination coverage for the completed three-dose primary series of the diphtheria-tetanus-pertussis (DTP3) vaccine was defined as the proportion of children in the annual birth cohort documented in the NIPIS as having received all three doses. This indicator aligns with the standard monitoring framework of the World Health Organization (23).

District-level socioeconomic and healthcare variables for Chongqing, including per capita disposable income, population density, and healthcare institution density, were obtained from the Chongqing Statistical Yearbook (2023) (19).

### Case definition

Pertussis cases were defined and classified according to the national diagnostic criteria effective at the time of case reporting (24–26). For this study, we included clinically diagnosed and laboratory-confirmed cases, while excluding suspected cases that did not meet these criteria. The major revisions during the study period are summarized below:

2013–2016: Case classifications included suspected, clinically diagnosed, and laboratory-confirmed cases based on the 2012 diagnostic criteria (24).

2017–2023: The case definition was updated in 2017 to incorporate polymerase chain reaction (PCR) as an acceptable laboratory confirmation method alongside culture (25).

2024: The criteria were substantially refined according to the Diagnosis and Treatment Protocol for Pertussis (2023 version) (26), which was formally released in late December 2023 and thus effective in the 2024 surveillance year. Key changes include:

- Refined clinical diagnostic criteria: A suspected case is classified as clinically diagnosed if it meets any of the following:

1. Peripheral blood leukocyte and lymphocyte counts are significantly elevated above the normal range for the corresponding age.
2. It presents with a paroxysmal spasmodic cough lasting ≥2 weeks and has a clear epidemiological link to a confirmed case (with onset intervals of 5–21 days).

- Enhanced laboratory confirmation: A suspected or clinically diagnosed case is confirmed if it meets any of the following:

1. Positive culture for *B. pertussis*.
2. Positive nucleic acid test for *B. pertussis*.
3. Seroconversion or a ≥ 4-fold increase in anti-PT-IgG titers between acute- and convalescent-phase serum samples (excluding infants vaccinated within the past year).

### Statistical analysis

Categorical variables are presented as numbers and percentages. These variables included sex, age group, urban or rural residence, population category, and the month and season of symptom onset. Age was categorized into the following groups for analysis: <3 months, 3–6 months, 6 months–1 year, 1–2 years, 2–3 years, 3–5 years, 5–10 years, 10–18 years, and ≥18 years. Seasons were defined as spring (March–May), summer (June–August), autumn (September–November), and winter (December–February). Group differences in categorical variables were assessed using the chi-square (χ^2^) test.

Crude and stratified pertussis notification rates were calculated using annual case counts and mid-year population estimates. Rates were computed for the entire population and further stratified by age group, sex, and district/county, using annual mid-year population data as denominators. Sex differences in pertussis incidence were quantified using Poisson regression models, which included the logarithm of the sex-specific population as an offset to account for differences in population size.

We analyzed temporal trends using Joinpoint Regression Software (version 4.9.1.0; National Cancer Institute, Bethesda, MD, United States) (27). The optimal number of joinpoints was determined using the Monte Carlo Permutation Test, in accordance with the standard methodology (28). Trends were characterized using the annual percent change (APC) for specific segments and the average annual percent change (AAPC) over a fixed interval, each presented with its 95% confidence interval (CI). Statistical significance was defined as a 95% *CI* that excluded zero. To assess the impact of the unprecedented surge in 2024, we first compared the overall AAPC for the full study period (2013–2024) against that of the pre-outbreak period (2013–2023). Subgroup analyses, which were conducted for the full period (2013–2024), were stratified by age and sex.

We performed an interrupted time series (ITSA) analysis to evaluate the impact of the revisions to the diagnostic criteria (29, 30). The models estimated the underlying pre-intervention trend, the immediate level change following the 2017 revision, the change in trend (slope) after the 2017 revision, and the immediate level change following the 2024 revision. Model coefficients were exponentiated to generate incidence rate ratios (IRRs) and 95% CIs.

Spatial analyses comprised three components: (1) Geographic mapping of county-level notification rates using ArcGIS (version 10.7; ESRI Inc.); (2) Assessment of spatial autocorrelation using global and local Moran’s I statistics in GeoDa (version 1.10), including Local Indicators of Spatial Association (LISA) analysis to identify significant spatial clusters (high-high, high-low, low-high, and low-low), visualized on LISA cluster maps (31); (3) Spatiotemporal cluster analysis using a discrete Poisson model within the prospective space–time scan statistic (SaTScan™ version 10.1.2) (32). The analysis utilized counties as the spatial unit and months as the temporal unit. The maximum spatial cluster size was constrained to 20% of the population at risk, a standard parameter that prioritizes the detection of focal, community-driven outbreaks (32, 33). The maximum temporal cluster size was set to 20% of the study duration (i.e., 2.4 months for our 12-month period), aligning with the natural history of pertussis outbreak cycles (34, 35). Statistical significance was evaluated using 999 Monte Carlo permutations. The cluster with the highest log-likelihood ratio (LLR) value was identified as the most likely cluster, and other significant clusters were classified as secondary (*p* < 0.05).

We fitted a multilevel linear mixed-effects model (36) to identify district-level factors associated with the pertussis notification rates. Model estimation was performed using the lmer() function from the lme4 package in R (37). Fixed effects comprised district-level covariates: vaccination coverage, per capita disposable income, population density, density of healthcare institutions, and binary indicators for the 2017 and 2024 revisions of the national diagnostic criteria. All continuous predictors were standardized to z-scores to enhance comparability of effect sizes. To account for the hierarchical data structure and non-independence of observations, we specified crossed random intercepts for district and year. The annual notification rate was natural-log-transformed to approximate normality, with a constant of 0.001 added to accommodate zero values—an established approach for log-transforming rate data containing zeros. Results are presented as coefficients (*β*) with 95% confidence intervals; for standardized continuous predictors, these represent the percentage change in notification rate per standard deviation increase, while for binary predictors they indicate percentage differences relative to the reference category.

To evaluate the robustness of our findings, we conducted a sensitivity analysis in which the pertussis notification rate was recalculated using only laboratory-confirmed cases as the numerator. Both the ITSA analysis and the multilevel model were then repeated using this revised rate.

All analyses were conducted using R software (version 4.0.2; R Foundation for Statistical Computing), except the joinpoint regression, spatial autocorrelation, and spatiotemporal scan statistics, for which specialized software was used as detailed above. A two-sided *p*-value <0.05 was considered statistically significant.

## Results

### Study population characteristics and seasonality

Between 2013 and 2024, 33,226 pertussis cases were notified in Chongqing, including 22,777 (68.55%) in 2024. The median patient age was 6 years (interquartile range [IQR]: 3–9 years; range: 1 day to 96 years). Case distribution varied significantly by year, sex, age group, urban/rural residence, occupation, and temporal characteristics (month and season of onset; all *p* < 0.05; Table 1). Males accounted for 51.6% (17,138/33,226) of cases. From 2013 to 2023, infants (aged <1 year) accounted for the largest proportion of cases (56.8%; 5,939/10,449). Within this group, infants aged <3 months accounted for 44.7% (2,658/5,939) of cases. A distinct epidemiological shift occurred in 2024, as children aged 5–10 years replaced infants as the group with the most cases (*n* = 13,868), with a case count 7.4 times the number reported in infants (*n* = 1,873; Table 1).

**Table 1:** Characteristics of notified pertussis cases in Chongqing, China, 2013–2024.

Characteristic	Total	2013	2014	2015	2016	2017	2018	2019	2020	2021	2022	2023	2024	Statistics	*P*-value
Sex														37.60	<0.001
Male	17,138 (51.6)	35 (59.3)	75 (45.5)	212 (57.0)	328 (53.3)	576 (55.0)	1,197 (55.0)	1,079 (51.3)	162 (52.3)	229 (58.0)	1,461 (52.5)	219 (51.4)	11,565 (50.8)		
Female	16,088 (48.4)	24 (40.7)	90 (54.5)	160 (43.0)	287 (46.7)	472 (45.0)	979 (45.0)	1,023 (48.7)	148 (47.7)	166 (42.0)	1,320 (47.5)	207 (48.6)	11,212 (49.2)		
Age group														-	<0.001*
<3 m	3,486 (10.5)	24 (40.7)	87 (52.7)	160 (43.0)	241 (39.2)	355 (33.9)	524 (24.1)	601 (28.6)	56 (18.1)	75 (19.0)	469 (16.9)	66 (15.5)	828 (3.6)		
3–6 m	2,058 (6.2)	9 (15.2)	49 (29.7)	92 (24.7)	121 (19.7)	210 (20.0)	383 (17.6)	392 (18.7)	39 (12.6)	33 (8.4)	230 (8.3)	36 (8.3)	464 (2.0)		
6 m–1 y	2,268 (6.8)	14 (23.7)	14 (8.5)	77 (20.7)	115 (18.7)	248 (23.7)	537 (24.7)	379 (18.0)	57 (18.4)	44 (11.1)	166 (6.0)	36 (8.5)	581 (2.7)		
1–2 y	1,984 (6.0)	4 (6.8)	7 (4.3)	28 (7.5)	90 (14.6)	136 (13.0)	356 (16.4)	279 (13.3)	55 (17.7)	44 (11.1)	115 (4.1)	22 (5.2)	848 (3.7)		
2–3 y	1,003 (3.0)	3 (5.1)	2 (1.2)	4 (1.1)	16 (2.6)	34 (3.2)	109 (5.0)	128 (6.1)	29 (9.4)	46 (11.6)	76 (2.7)	19 (4.5)	537 (2.4)		
3–5 y	2,882 (8.7)	1 (1.7)	3 (1.8)	8 (2.2)	19 (3.1)	43 (4.1)	157 (7.2)	177 (8.4)	45 (14.5)	78 (19.8)	311 (11.2)	52 (12.2)	1,988 (8.7)		
5–10 y	15,584 (46.9)	2 (3.4)	2 (1.2)	2 (0.5)	13 (2.1)	16 (1.5)	101 (4.6)	130 (6.2)	27 (8.7)	65 (16.5)	1,190 (42.8)	168 (39.4)	13,868 (60.9)		
10–18 y	2,581 (7.8)	2 (3.4)	0 (0.0)	1 (0.3)	0 (0.0)	5 (0.5)	7 (0.3)	15 (0.7)	2 (0.6)	8 (2.0)	189 (6.8)	19 (4.5)	2,333 (10.2)		
≥18 y	1,380 (4.1)	0 (0.0)	1 (0.6)	0 (0.0)	0 (0.0)	1 (0.1)	2 (0.1)	1 (0.0)	0 (0.0)	2 (0.5)	35 (1.2)	8 (1.9)	1,330 (5.8)		
Residence														687.04	<0.001
Urban	26,805 (80.7)	26 (44.1)	105 (63.6)	248 (66.7)	421 (68.5)	758 (72.3)	1,575 (72.4)	1,551 (73.8)	178 (57.4)	264 (66.8)	2,287 (82.2)	334 (78.4)	19,058 (83.7)		
Rural	6,421 (19.3)	33 (55.9)	60 (36.4)	124 (33.3)	194 (31.5)	290 (27.7)	601 (27.6)	551 (26.2)	132 (42.6)	131 (33.2)	494 (17.8)	92 (21.6)	3,719 (16.3)		
Population category														-	<0.001*
Preschool Children	11,524 (34.7)	54 (91.5)	160 (97.0)	363 (97.6)	586 (95.3)	993 (94.8)	1,945 (89.4)	1,825 (86.8)	248 (80.0)	254 (64.3)	1,125 (40.4)	190 (44.6)	3,781 (16.6)		
Students	11,791 (35.5)	3 (5.1)	2 (1.2)	2 (0.5)	4 (0.6)	14 (1.3)	59 (2.7)	69 (3.3)	10 (3.2)	36 (9.1)	872 (31.4)	139 (32.6)	10,581 (46.5)		
Other	9,911 (29.8)	2 (3.4)	3 (1.8)	7 (1.9)	25 (4.1)	41 (3.9)	172 (7.9)	208 (9.9)	52 (16.8)	105 (26.6)	784 (28.2)	97 (22.8)	8,415 (36.9)		
Month														-	<0.001*
January	597 (1.8)	3 (5.1)	2 (1.2)	8 (2.0)	33 (5.4)	33 (3.1)	47 (2.2)	75 (3.6)	37 (11.9)	33 (8.4)	90 (3.2)	14 (3.3)	222 (1.0)		
February	927 (2.8)	5 (8.5)	6 (3.6)	26 (7.0)	77 (12.5)	53 (5.1)	109 (5.0)	132 (6.3)	52 (16.8)	28 (7.1)	148 (5.3)	14 (3.3)	277 (1.2)		
March	2,054 (6.2)	2 (3.4)	11 (6.7)	34 (9.1)	56 (9.1)	74 (7.1)	244 (11.2)	196 (9.3)	36 (11.6)	31 (7.9)	252 (9.1)	38 (8.9)	1,080 (4.7)		
April	5,332 (16.0)	5 (8.5)	17 (10.3)	39 (10.5)	48 (7.8)	75 (7.2)	308 (14.1)	258 (12.3)	24 (7.7)	25 (6.3)	368 (13.2)	46 (10.8)	4,119 (18.1)		
May	7,404 (22.3)	6 (10.2)	24 (14.6)	30 (8.1)	71 (11.5)	105 (10.0)	261 (12.0)	277 (13.2)	13 (4.2)	25 (6.3)	551 (19.8)	27 (6.3)	6,014 (26.4)		
June	6,886 (20.7)	7 (11.9)	17 (10.3)	56 (15.1)	73 (11.9)	168 (16.0)	253 (11.6)	281 (13.4)	17 (5.5)	28 (7.1)	581 (20.9)	42 (9.9)	5,363 (23.5)		
July	5,093 (15.3)	15 (25.4)	34 (20.6)	57 (15.3)	97 (15.8)	154 (14.7)	391 (18.0)	303 (14.4)	11 (3.6)	38 (9.6)	345 (12.4)	32 (7.5)	3,616 (15.9)		
August	2,477 (7.5)	9 (15.1)	28 (17.0)	48 (12.9)	70 (11.4)	154 (14.7)	300 (13.8)	269 (12.8)	7 (2.3)	46 (11.6)	225 (8.1)	33 (7.7)	1,288 (5.7)		
September	977 (2.9)	1 (1.7)	13 (7.9)	13 (3.5)	36 (5.9)	90 (8.6)	110 (5.1)	135 (6.4)	19 (6.1)	17 (4.3)	97 (3.5)	32 (7.5)	414 (1.8)		
October	585 (1.8)	4 (6.8)	4 (2.4)	20 (5.4)	19 (3.1)	68 (6.5)	68 (3.1)	68 (3.2)	34 (11.0)	18 (4.6)	78 (2.8)	39 (9.2)	165 (0.7)		
November	382 (1.2)	1 (1.7)	7 (4.2)	14 (3.8)	15 (2.4)	36 (3.4)	45 (2.1)	52 (2.5)	27 (8.7)	32 (8.1)	28 (1.0)	36 (8.5)	89 (0.4)		
December	512 (1.5)	1 (1.7)	2 (1.2)	27 (7.3)	20 (3.2)	38 (3.6)	40 (1.8)	56 (2.6)	33 (10.6)	74 (18.7)	18 (0.7)	73 (17.1)	130 (0.6)		
Season														-	<0.001*
Spring (March–May)	14,790 (44.5)	13 (22.0)	52 (31.5)	103 (27.7)	175 (28.5)	254 (24.2)	813 (37.4)	731 (34.8)	73 (23.5)	81 (20.5)	1,171 (42.1)	111 (26.1)	11,213 (49.2)		
Summer (June–August)	14,456 (43.5)	31 (52.5)	79 (47.9)	161 (43.3)	240 (39.0)	476 (45.4)	944 (43.4)	853 (40.6)	35 (11.3)	112 (28.4)	1,151 (41.4)	107 (25.1)	10,267 (45.1)		
Autumn (September–November)	1,944 (5.9)	6 (10.2)	24 (14.5)	47 (12.6)	70 (11.4)	194 (18.5)	223 (10.2)	255 (12.1)	80 (25.8)	67 (17.0)	203 (7.3)	107 (25.1)	668 (2.9)		
Winter (December–February)	2,036 (6.1)	9 (15.3)	10 (6.1)	61 (16.4)	130 (21.1)	124 (11.8)	196 (9.0)	263 (12.5)	122 (39.4)	135 (34.2)	256 (9.2)	101 (23.7)	629 (2.8)		

*Fisher’s exact test; Seasons: Spring (March–May), Summer (June–August), Autumn (September–November), Winter (December–February).

**Figure 2:**
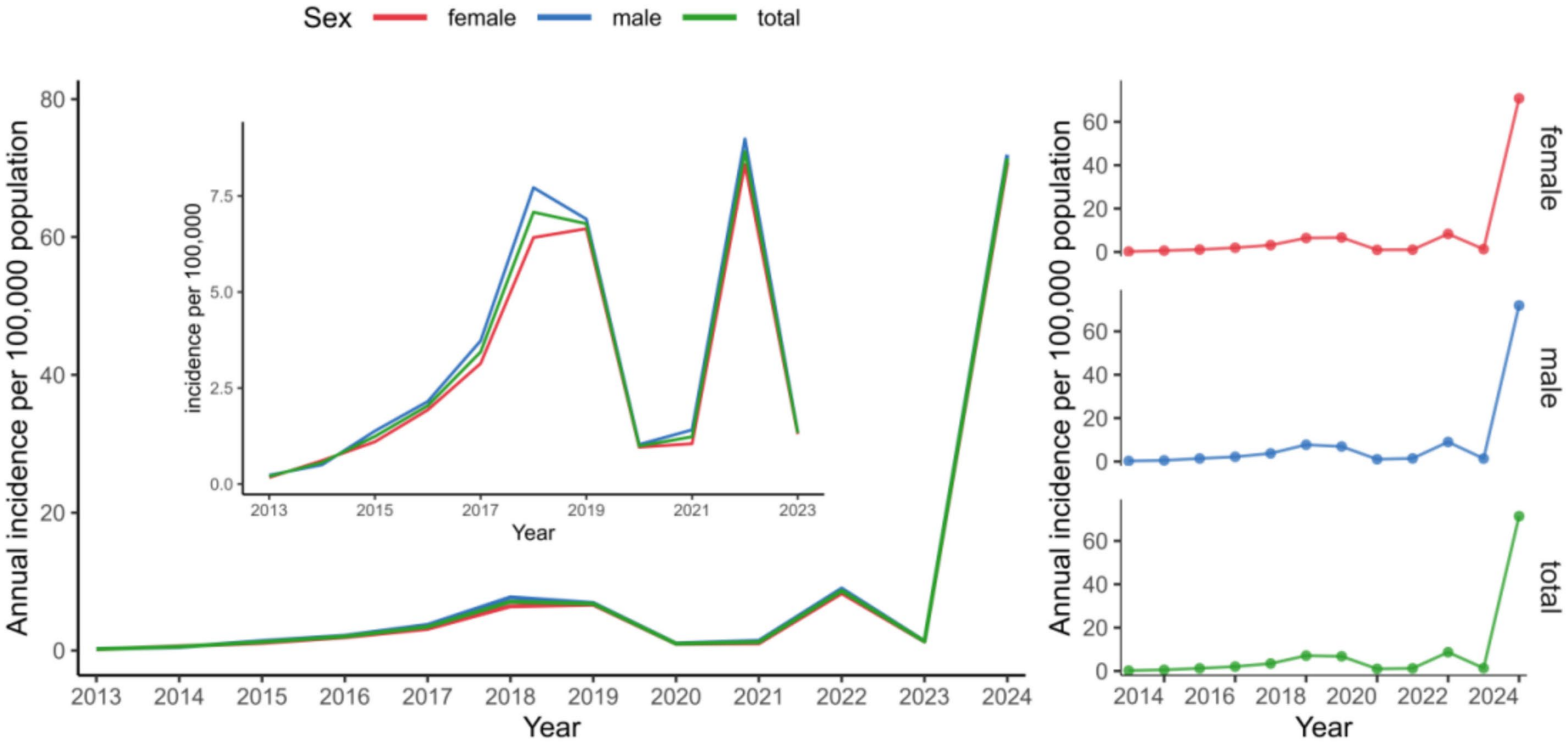
Sex-stratified temporal dynamics of pertussis notification rate in Chongqing, China, 2013–2024.

**Figure 3:**
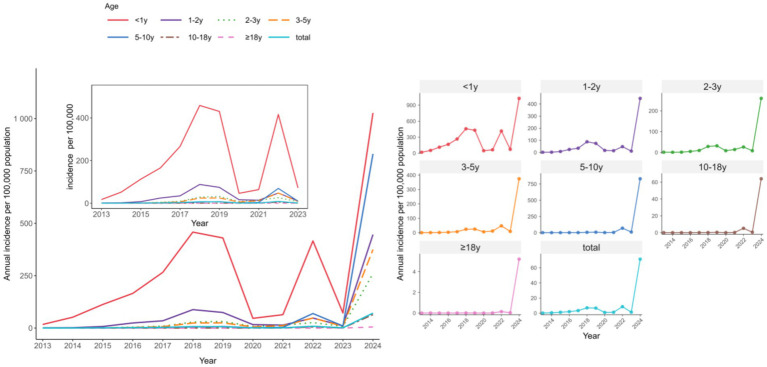
Age-stratified temporal dynamics of pertussis notification rate in Chongqing, China, 2013–2024.

A consistent pattern of higher pertussis notification rates among males compared with females was observed throughout the study (Figure 2). Throughout the 2013–2024 surveillance period, infants consistently exhibited the highest pertussis notification rates. The rate among infants (aged <1 year) increased from 17.25 per 100,000 person-years in 2013 to 1,027.14 in 2024. In 2024, substantial increases in notification rates were observed across all age groups, with children aged 5–10 years showing the second-highest rate (831.69 per 100,000 person-years; Figure 3).

Surveillance data revealed consistent spring–summer seasonality from 2013 to 2019, with 87.80% of cases occurring between March and August. The incidence peaked in May (23.73% of annual cases) and June (21.22%). This pattern reversed during the 2020–2021 period, when 65.60% of cases were reported between December and February, peaking in December (15.18%) and February (11.35%). The typical spring–summer pattern was re-established from 2022 to 2024.

**Figure 4:**
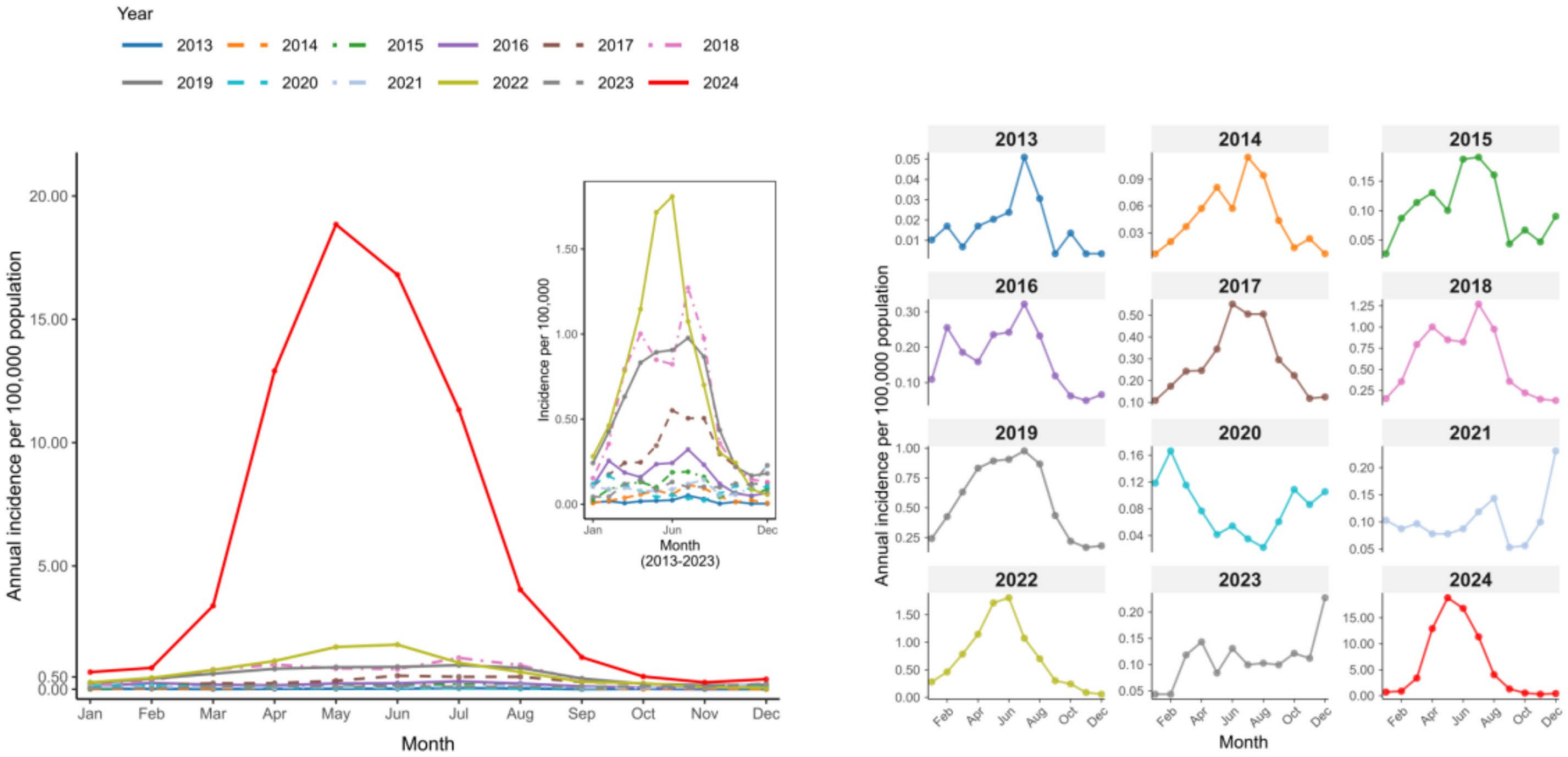
Month-stratified temporal dynamics of pertussis notification rate in Chongqing, China, 2013–2024.

**Figure 5:**
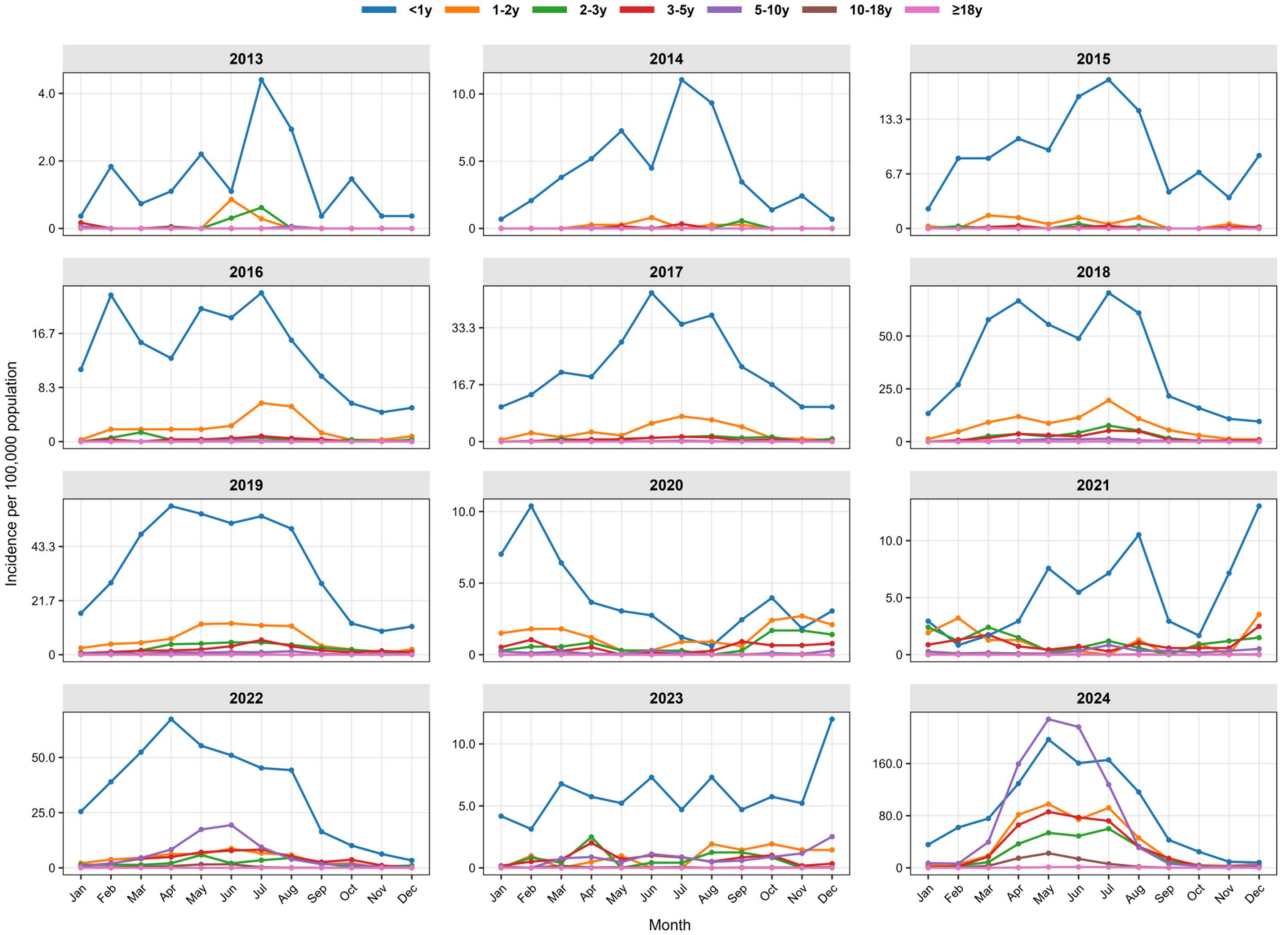
Age-specific seasonality of monthly pertussis incidence in Chongqing, China, 2013–2024.

Distinct age-specific seasonal patterns were identified between 2013 and 2024 (Figures 4, 5). Infants (<1 year) consistently had the highest annual rates, demonstrating a recurring summer peak (June–August). From 2021 onwards, school-aged children (5–10 years) showed a dramatic increase in incidence, forming intense spring–summer epidemics (February/March–July). Meanwhile, adolescents (10–18 years) and adults (≥18 years) exhibited minimal transmission before 2022, after which both groups experienced a sustained increase and developed visible seasonal peaks.

Age-stratified Poisson regression analysis revealed distinct sex differences in pertussis notification rates across age groups (Supplementary Table 1). The notification rate was significantly higher in males than in females among infants aged <1 year (IRR = 1.11, 95% CI: 1.06–1.16, *p* < 0.001). Conversely, the notification rate was significantly higher in females among adults (≥18 years; IRR = 0.64, 95% CI: 0.57–0.71, *p* < 0.001) and preschool-aged children (3–5 years; IRR = 0.88, 95% CI: 0.82–0.95, *p* < 0.01). No statistically significant sex differences were observed in the other childhood age groups (1–2, 2–3, 5–10, and 10–18 years).

### Temporal trends

#### Joinpoint regression analysis

The long-term trend in pertussis notification rates during the pre-outbreak period (2013–2023) was not statistically significant. Joinpoint regression identified a change point in 2017, segmenting the period into a phase of non-significant increase from 2013 to 2017 (APC = 113.41, 95% CI: −17.17 to 449.85) followed by a phase of non-significant decline from 2017 to 2023 (APC = -14.15, 95% CI: −48.23 to 42.38).

In stark contrast, the analysis including the outbreak year 2024 revealed a significant overall increasing trend from 2013 to 2024, with an AAPC of 32.16% (95% CI: 5.40 to 65.72). This significant overall trend is overwhelmingly driven by the unprecedented surge in cases observed in 2024. A comparable trend was observed in laboratory-confirmed cases (AAPC = 30.31, 95% CI: 1.17 to 67.84). Significant increases were also evident in both females (AAPC = 32.75, 95% CI: 5.52 to 67.00) and males (AAPC 31.81, 95% CI: 5.29 to 65.01).

For the full 2013–2024 period, age-stratified analyses showed significant increasing trends across all age groups except infants under 1 year, who exhibited a non-significant increase (AAPC = 17.86%; 95% CI: −3.97 to 44.66). The most pronounced increase was identified in children aged 5–10 years (AAPC = 94.91%; 95% CI: 55.18 to 144.81). Adolescents aged 10–18 years also exhibited a high AAPC of 89.32%, although with a wider confidence interval (95% CI: 36.38 to 162.82). These were followed by adults ≥18 years (AAPC = 79.86%; 95% CI: 25.47 to 157.82) and younger children aged 3–5 years (AAPC = 64.00%; 95% CI: 33.53 to 101.42%), 2–3 years (AAPC = 48.39%; 95% CI: 22.28 to 80.07), and 1–2 years (AAPC = 36.55%; 95% CI: 7.80 to 72.97). Interrupted Time Series Analysis (ITSA).

The interrupted time series analysis (ITSA) evaluated the impact of diagnostic criteria revisions on pertussis notification rates (Table 2). The 2017 revision was not associated with an immediate level change (all cases: *IRR* = 1.10, 95% *CI*: 0.69–1.77, *p =* 0.685; laboratory-confirmed cases: *IRR* = 0.62, 95% *CI*: 0.37–1.04, *p =* 0.068) but significantly attenuated the pre-existing upward trend (all cases: *IRR* = 0.44, 95% *CI*: 0.37–0.53, *p* < 0.001; laboratory-confirmed cases: *IRR* = 0.39, 95% *CI*: 0.32–0.48, *p* < 0.001).

**Table 2:** Impact of diagnostic guideline revisions on pertussis notification rates.

Parameter	All cases		Laboratory-confirmed cases only	
	IRR (95% CI)	*P*-value	IRR (95% CI)	*P*-value
2017 diagnostic criteria revision				
Level change	1.10 (0.69, 1.77)	0.685	0.62 (0.37, 1.04)	0.068
Trend change	0.44 (0.37, 0.53)	<0.001	0.39 (0.32, 0.48)	<0.001
2024 diagnostic criteria revision				
Level change	26.46 (17.52, 39.95)	<0.001	62.13 (39.85, 96.87)	<0.001

IRR: incidence rate ratio.

**Figure 6:**
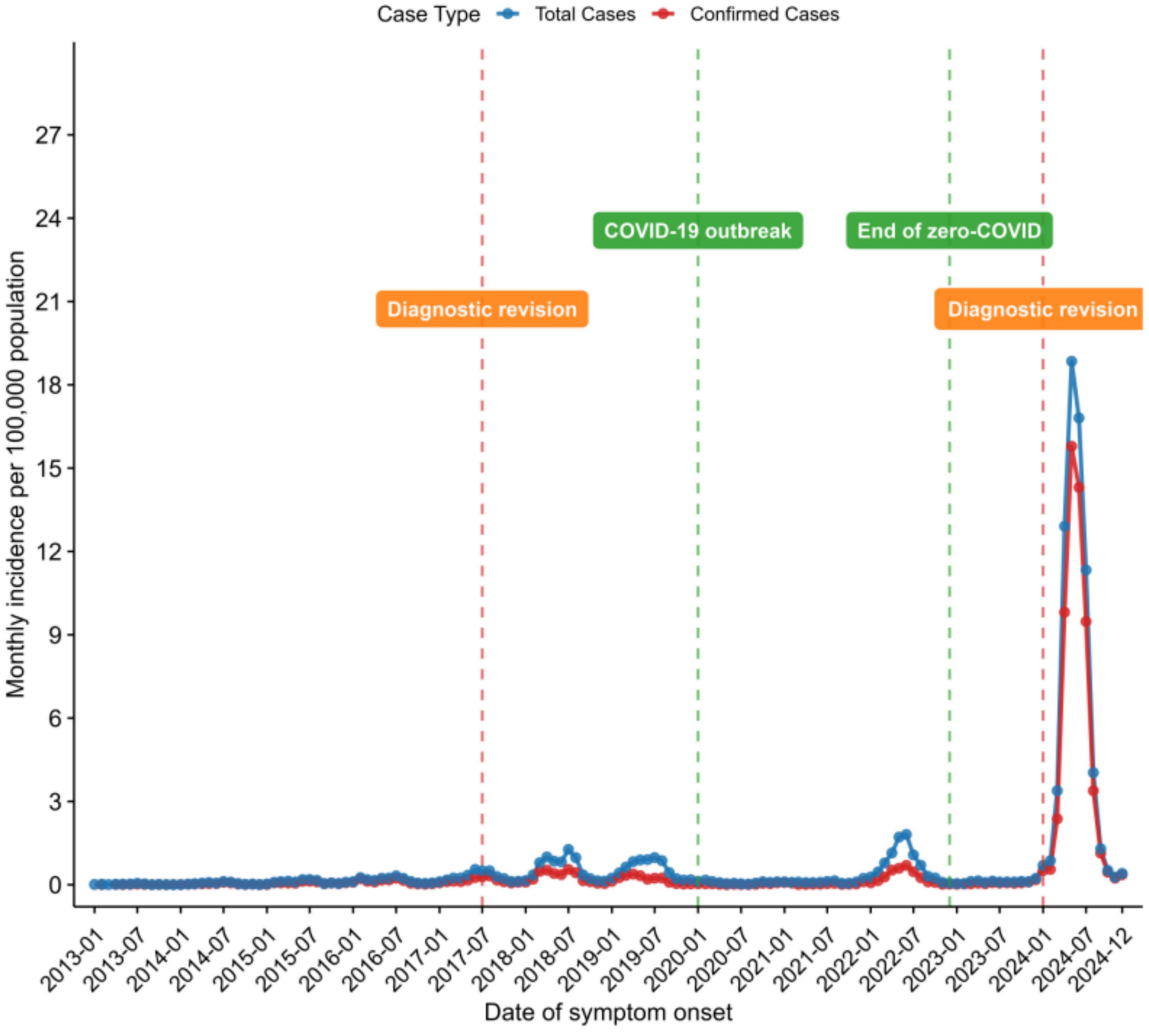
Annual pertussis notification rates in Chongqing, China, 2013–2024.

Conversely, the 2024 revision was associated with an immediate and substantial increase in notification rates, with IRRs of 26.46 (95% CI: 17.52–39.95) for all cases and 62.13 (95% CI: 39.85–96.87) for laboratory-confirmed cases (both *p* < 0.001). These findings are summarized in Figure 6.

### Spatial and spatiotemporal clustering of pertussis

#### Spatiotemporal distribution

Analysis of the spatiotemporal distribution identified higher pertussis notification rates in 2018, 2019, 2022, and 2024 across Chongqing districts/counties (Supplementary Figure 1).

#### Spatial autocorrelation analysis

Global spatial autocorrelation analysis demonstrated statistically significant positive spatial autocorrelation from 2014 to 2019 and in 2022 (Moran’s I range: 0.024–0.572; all *p* < 0.05) (Supplementary Table 2). LISA analysis identified statistically significant high-high clusters of pertussis. From 2014 through 2019, these clusters were primarily concentrated in the central urban area; they reemerged in 2021 and 2022 and exhibited a limited spatial extent in 2024 (Supplementary Figure 2).

#### Spatiotemporal cluster analysis

A most likely pertussis cluster was detected in each year of the study period. These clusters demonstrated distinct seasonal patterns. From 2013 to 2018, they consistently occurred during summer months (June to August). In 2019 and 2022, clusters were identified from May to June. A notable temporal shift was observed during the 2020–2021 period, with clusters detected from November to December 2020 and from January to February 2021.

In 2023, the cluster occurred from November to December, and in 2024, it occurred from April to May. Geographically, the most likely pertussis clusters were centered in the following districts and counties: Tongnan District (2013); Jiangbei District (2014, 2018); Yubei District (2015, 2016, 2019, 2022); Yuzhong District (2017); Dianjiang County (2020, 2021); Jiangjin District (2023); and Dadukou District (2024). These pertussis clusters exhibited significantly elevated relative risks, with RR values ranging from 3.43 (Yubei District, 2019; 95% *CI*: 2.77–4.26) to 47.66 (Tongnan District, 2013; 95% *CI*: 0.78–2907.27) (Supplementary Table 3; Supplementary Figure 3).

#### Factors associated with notification rates

Multilevel regression analysis identified several factors significantly associated with notification rates. A strong positive association was observed between higher income levels and the pertussis notification rate (*β* = 0.56, 95% CI: 0.27–0.84, *p* < 0.001). Similarly, the 2024 diagnostic criteria revision was associated with a substantial increase in notification rates (*β =* 5.52, 95% CI: 1.48–9.55, *p =* 0.013). Conversely, no statistically significant associations were found between the pertussis notification rate and vaccination coverage, population density, number of healthcare institutions, or the 2017 revision of diagnostic criteria. The findings remained consistent in a sensitivity analysis restricted to laboratory-confirmed cases, confirming the robustness of the primary results (Table 3).

**Table 3:** Ecological analysis of district-level factors associated with pertussis notification rates.

Parameter	Model 1		Model 2	
	β (95% CI)	*P*-value	β (95% CI)	*P*-value
Fixed effects				
Intercept	−1.52 (−3.33, 0.29)	0.089	−2.15 (−4.42, 0.12)	0.061
Vaccination coverage rate	0.01 (−0.18, 0.19)	0.938	−0.04 (−0.25, 0.17)	0.725
Population density	−0.11 (−0.37, 0.15)	0.410	−0.20 (−0.48, 0.09)	0.174
Number of health institutions	−0.09 (−0.33, 0.14)	0.431	0.01 (−0.25, 0.27)	0.914
Income	0.56 (0.27, 0.84)	<0.001	0.57 (0.25, 0.88)	<0.001
Diagnostic criteria: 2017 revision	1.89 (−0.37, 4.15)	0.091	1.13 (−1.71, 3.97)	0.393
Diagnostic criteria: 2024 revision	5.52 (1.48, 9.55)	0.013	5.87 (0.80, 10.94)	0.028
Random effects				
County variance (SD)	0.16 (0.40)		0.16 (0.40)	
Year variance (SD)	2.47 (1.57)		3.91 (1.98)	
Residual variance (SD)	2.97 (1.72)		4.04 (2.01)	

Analysis compares notification rates under two case definitions. Model 1 includes all notified cases; Model 2 is restricted to laboratory-confirmed cases to assess robustness.

## Discussion

Our 12-year longitudinal analysis reveals a critical epidemiological shift in pertussis transmission dynamics in Chongqing. Over the 12-year study period (2013–2024), infants (aged <1 year) consistently had the highest notification rates, which surged from 17.25 to 1027.14 per 100,000 person-years. Notably, infants aged <3 months accounted for 25% of all reported cases during the 2013–2023 study period, a trend consistent with surveillance data from multiple provinces across China (17, 18).

This vulnerability arose from China’s pre-2025 immunization schedule, under which infants aged <3 months had no vaccine-induced immunity, while those aged 3–12 months had incomplete protection during the primary series (38). Furthermore, the underrecognition of adult pertussis due to widespread self-medication and low clinical suspicion creates a hidden reservoir of infection, elevating the risk of household transmission to young infants (39). As a result of this heightened exposure risk and their own susceptibility, the majority of severe cases occur in infants aged < 3 months, as emphasized by Kandeil et al. (3), highlighting a critical window of susceptibility before vaccine eligibility. Maternal Tdap vaccination during pregnancy is therefore a vital strategy for bridging this immunity gap (40). A US case–control study demonstrated that prenatal Tdap vaccination administered between 27 and 36 weeks of gestation was 92.5% effective (95% CI: 38.5–99.1) in preventing pertussis in infants <2 months (41). Recognizing its value, over 40 countries have now implemented recommendations for maternal Tdap immunization, albeit with variations in the recommended gestational window (3). In response, China has adjusted its national immunization schedule, effective in 2025, which advances the first DTaP dose from 3 to 2 months of age and introduces a booster dose at 6 months old (22). While China has not yet introduced maternal Tdap immunization, this schedule revision represents a significant step towards reducing the vulnerability of young infants.

Our data strongly suggest that school-aged children (5–10 years) became the core transmitters driving community spread. This is supported by their pivotal role in initiating intense spring–summer epidemics from 2021 onward, culminating in 2024 when they constituted 60.89% of all reported cases—7.4 times the case count in infants (42). The subsequent emergence of distinct seasonal peaks in adolescents and adults after 2022 demonstrates the expansion of transmission from this school-aged reservoir, marking the establishment of sustained community-wide transmission chains (43). Our analysis revealed distinct age-dependent sex differences in pertussis incidence. A significant male bias was observed in infants under 1 year (IRR = 1.11, 95% CI: 1.06–1.16), which contrasts with the female bias found in adults (IRR = 0.64, 95% CI: 0.57–0.71) and preschool-aged children (3–5 years; IRR = 0.88, 95% CI: 0.82–0.95). This pattern of highest male susceptibility in infancy aligns with global epidemiological data showing the greatest heterogeneity in sex-specific incidence during the first year of life (44). The biological plausibility of this finding is supported by evidence that male infants have a well-documented increased susceptibility to respiratory infections, likely due to sex-based differences in early immune maturation (45).

The substantial increase in reported pertussis cases in 2024, particularly among school-aged children, warrants careful interpretation. Our ITSA identified a significant level change coinciding with the 2024 diagnostic criteria revision. The observed pattern, with school-aged children (5–10 years) constituting the majority of cases, while infants exhibit the highest notification rate, suggests that school-aged children now represent a key reservoir for *B. pertussis* transmission in Chongqing. However, it is critical to interpret this surge as resulting from both substantially enhanced surveillance sensitivity and a genuine underlying increase in pertussis transmission. This genuine increase may have been exacerbated by an accumulation of susceptible individuals during the COVID-19 pandemic, as evidenced by the pre-existing upward trends in these age groups.

Three strands of evidence converge to support this interpretation of surveillance enhancement superimposed on a genuine transmission increase. Primarily, the immediate and substantial surge in notifications, particularly for laboratory-confirmed cases, corresponds precisely with the implementation of the revised criteria. Furthermore, the epidemiological dynamics before 2024, characterized by an initial non-significant increase (APC = 113.41%, 2013–2017) followed by a period of non-significant decrease (APC = -14.15%, 2017–2023) in pertussis notification rate, indicate that underlying transmission was active and fluctuating, rather than following a stable or consistently declining trajectory. Finally, the characteristic shift in the age distribution in 2024 aligns with the specific enhancements to the diagnostic criteria aimed at improving detection in older age groups. While we cannot definitively quantify the relative contributions of improved detection versus factors such as the accumulation of susceptibles, the evidence strongly suggests that the diagnostic revision revealed a substantial and growing disease burden that was previously under-ascertained.

The pronounced increase in laboratory-confirmed cases is consistent with established knowledge that enhancements in diagnostic sensitivity can lead to improved case detection (46). This context is particularly relevant given that the infrastructure for molecular diagnostics, widely established for SARS-CoV-2 testing, is readily adaptable for the detection of other respiratory pathogens like *B. pertussis* (47). Furthermore, the sustained upward trajectory in cases aligns with the well-documented global resurgence of pertussis, which is largely attributed to the waning of immunity following pertussis vaccination (48, 49). This pattern of resurgence is not unique to Chongqing but reflects broader epidemiological trends reported in national surveillance data from China (50, 51) and aligns with global trends of the resurgence of pertussis, driven by age-group shifts (6). This epidemiological transition is likely driven by waning immunity following childhood vaccination. A recent study attributed China’s resurgence of pertussis largely to this demographic transition, characterized by a rising proportion of cases among older children (52).

Beyond the shifting age distribution, we observed a significant overall resurgence of pertussis in Chongqing from 2013 to 2024, with notification rates increasing from 0.20 to 71.38 per 100,000 person-years (AAPC = 32.16%). The parallel increase in laboratory-confirmed cases (AAPC = 30.31%) indicates an underlying rise in disease transmission, while the dramatic surge in 2024 likely represents the combined effect of this genuine resurgence and substantially enhanced surveillance sensitivity through the diagnostic criteria revision. This upward trend reflects the widely recognized resurgence of pertussis throughout China (6, 53).

Multiple interconnected mechanisms likely contribute to the observed resurgence of pertussis in Chongqing. Foremost is the waning of immunity following both vaccination and natural infection (2, 51, 54). Protection from aPVs wanes at an estimated rate of 9.6% (95% *CI*: 8.6–10.6) per year after completion of the primary series (55), substantially expanding the susceptible population over time (51, 54, 56). A systematic review confirmed this progressive waning, with the relative risk of infection increasing from 0.15 (95% CI: 0.09–0.26) at <1 year post-vaccination to 0.41 (95% CI: 0.30–0.56) at 5–7 years (57). Additionally, although the transition from whole-cell (DTwP) to DTaP vaccines reduced adverse effects, it may have contributed to resurgence due to less durable immunity (5). Furthermore, genomic evolution of *B. pertussis* under acellular vaccine pressure may also facilitate pathogen re-emergence. This evolution is characterized by emerging allelic variations that enhance immune evasion (58).

The patterns we observed in our age-stratified analysis are consistent with these resurgence mechanisms. Our analysis identified the most rapid increases in notification rates in school-aged children (5–10 years: *AAPC =* 94.91%) and adolescents (10–18 years: *AAPC* = 89.32%). Similar trends have also been observed in previous studies (57, 59). While we did not find direct evidence supporting waning immunity as a driver within our model, the observed trends are compatible with this established mechanism. However, the extraordinary magnitude of the surge in 2024 coincides temporally with the implementation of revised diagnostic criteria, suggesting that enhanced case detection substantially contributed to the observed increase. The legacy effect of historical vaccination practices, as posited by Domenech de Cellès et al. (60), whereby widespread whole-cell vaccination reduced natural immune boosting, may serve as an additional contributing factor. In China, the DTaP antigen combination forms the basis of the national immunization program. It is administered both as a standalone DTaP vaccine and as a component in multivalent vaccines, which may also include inactivated poliovirus (IPV) and *Haemophilus influenzae* type b (Hib) antigens (61). Compared to the pre-2025 schedule, China’s 2025 revision advances the first DTaP dose from 3 to 2 months, delays the third primary dose from 5 to 6 months, extends the interdose interval from one to two months, and replaces the DT booster at 6 years of age with a DTaP vaccine (22). Our interrupted time-series analysis reveals the profound impact of revisions to China’s national diagnostic criteria on pertussis surveillance data in Chongqing. The attenuated notification rate following the 2017 guideline implementation (25), which mandated laboratory confirmation through serological testing and PCR, likely reflects improved diagnostic specificity that reduced false-positive reporting. Most strikingly, the immediate 62.1-fold surge in laboratory-confirmed cases following the 2024 revision corresponds directly to the 2023 national protocol (26) that introduced clinical epidemiological criteria and enhanced molecular detection capacity. This magnitude of increase underscores the profound impact of surveillance changes, though it likely captures a combination of previously undetected cases and genuine epidemiological growth.

These divergent trends demonstrate that both surveillance changes and genuine epidemiological shifts can profoundly influence observed pertussis patterns, highlighting the importance of considering multiple factors when interpreting surveillance data. This underscores the critical importance of accounting for changes in diagnostic policy when interpreting pertussis trends over time in China (25, 26).

Our multilevel model identified higher district-level income as an independent predictor of elevated pertussis notification rates (*β* = 0.56, *p* < 0.001), consistent with literature linking socioeconomic status (SES) to disease surveillance sensitivity (2). Specifically, higher-SES populations have greater access to primary care and fewer financial barriers to seeking care when symptomatic, factors that enhance the likelihood of pertussis diagnosis and subsequent reporting (62). Furthermore, the absence of a significant association between aggregate vaccination coverage and notification rates at the district level likely reflects the limited variability in coverage (which was consistently high across districts) and the ecological nature of this analysis, which cannot account for individual-level protection. This ecological finding, which does not measure individual-level vaccine effectiveness and should not be construed as evidence against it, is consistent with the broader evidence that high childhood coverage alone cannot prevent resurgences (2).

Pertussis exhibited a consistent seasonal pattern in Chongqing, with annual peaks in May–June during 2013–2019, 2022, and 2024, but this pattern was disrupted in 2020–2021, likely due to COVID-19-related non-pharmaceutical interventions (NPIs) that suppressed respiratory pathogen transmission (63). This seasonal profile aligns with observations from other regions in China (18), France (8), Germany (64), and Vietnam (65). However, pertussis seasonality remains ambiguous: in winter and spring, high prevalence of influenza, mycoplasma, and respiratory syncytial virus infections leads to widespread cough symptoms, which overlap with those of atypical pertussis and complicate its diagnosis and reporting, potentially resulting in underreporting (8, 48). Pertussis cases are often misdiagnosed as other respiratory viral infections during winter, contributing to unclear seasonality (48).

Pertussis notification rates in Chongqing from 2013 to 2024 exhibited a non-random spatial distribution at the district/county level. Global spatial autocorrelation analysis confirmed a non-random, clustered distribution of pertussis cases across Chongqing. Spatial hotspots demonstrated both temporal dynamism and persistent concentration in the Central Urban Area and adjacent regions throughout the study period. These findings are consistent with the spatial patterns of pertussis notification rates, which were consistently higher in the Central Urban Area. This clustering may be attributed to the region’s role as the political and transportation hub of the municipality, supporting high population densities and substantial inbound migration (20, 66). Superior healthcare infrastructure, greater accuracy and sensitivity of surveillance systems, and well-developed transportation networks likely contribute to higher case detection rates in these areas (13). Furthermore, factors such as economic status, geographic location, and frequent population movement likely contribute to greater pertussis clustering in the Central Urban Area.

Spatiotemporal analysis further confirmed that the most likely cluster of pertussis cases was consistently located within the Central Urban Area, reinforcing its status as a high-risk region. This pattern is consistent with the typical diffusion of infectious diseases, which often emerge initially in urban centers before spreading to outlying areas (17, 67). The extreme *RR* estimates observed in some clusters should be interpreted with caution. While statistically significant, these implausibly high values are likely methodological artifacts arising from the analysis of areas with low expected case counts (33, 68, 69). Thus, statistical significance reliably identifies foci of elevated transmission, but the *RR* magnitude is best interpreted as a qualitative, rather than precise quantitative, indicator of elevated risk. The concentration of population, high-frequency human interaction (19, 20, 66), and superior transportation networks in this core area provide ideal conditions for pertussis transmission. Furthermore, the aggregation of advanced medical facilities likely enhances surveillance sensitivity through more frequent testing, potentially increasing the recorded notification rate in this area. The epidemiology of pertussis is shaped by a multifaceted confluence of these social and biological factors (2, 70).

Our study offers a comprehensive 12-year longitudinal analysis of pertussis epidemiology in Chongqing, one of China’s four major municipalities. To our knowledge, it represents one of the most detailed integrations of demographic evolution, spatiotemporal clustering, and vaccination coverage dynamics within a dense urban setting in China, providing empirical evidence that aligns with the national immunization schedule revision effective in 2025 (22). A key finding from this detailed analysis is the notable shift in age distribution: although infants <1 year were the most affected group between 2013 and 2023, school-aged children (5–10 years) emerged as the predominant group in 2024, bearing a burden 7.4 times that of infants. This clear transition—alongside the persistently high notification rate among very young infants—offers robust, evidence-based justification for accelerating the development and rollout of a maternal Tdap immunization program to protect newborns during early infancy.

This study has several limitations that should be considered when interpreting the findings. First, as with all analyses based on passive surveillance data, our observed pertussis notification rates are likely subject to significant underascertainment. Recent evidence from active surveillance studies in China demonstrates that community-based incidence estimates substantially exceed rates reported through passive systems, confirming that passive surveillance captures only a fraction of the true disease burden, particularly among older children and adults (71). The probability of case identification is influenced by multiple factors that exhibit geographic and age-related variation, including healthcare-seeking behavior, access to diagnostic services, and testing practices (71, 72). Second, although we employed age-stratified analyses to account for demographic changes, the absence of nationally representative standard population weights precluded calculation of age-standardized rates. This limitation is partially mitigated by our age-specific analytical approach, which enables valid comparisons within specific age groups.

In conclusion, our analysis revealed a substantial resurgence of pertussis in Chongqing from 2013 to 2024, characterized by a pivotal epidemiological shift in 2024, when school-aged children (5–10 years) became the largest case group while infants maintained the highest notification rates. This finding underscores the critical need to implement seroepidemiological surveillance to directly measure population immunity and rationally guide the development of targeted interventions.

## Data Availability

The raw data supporting the conclusions of this article will be made available by the authors, without undue reservation, subject to the constraints of applicable laws and policies.
