# Supplementary material for: Epidemiological and spatial–temporal clustering characteristics of pertussis in Southwest China, 2013–2024
Source: Front Public Health. 2026 Jan 12;13:1620429. doi: 10.3389/fpubh.2025.1620429 (PMC12832240; doi:10.3389/fpubh.2025.1620429)
Supplement: Supplementary file 1 [file Table_1.docx]

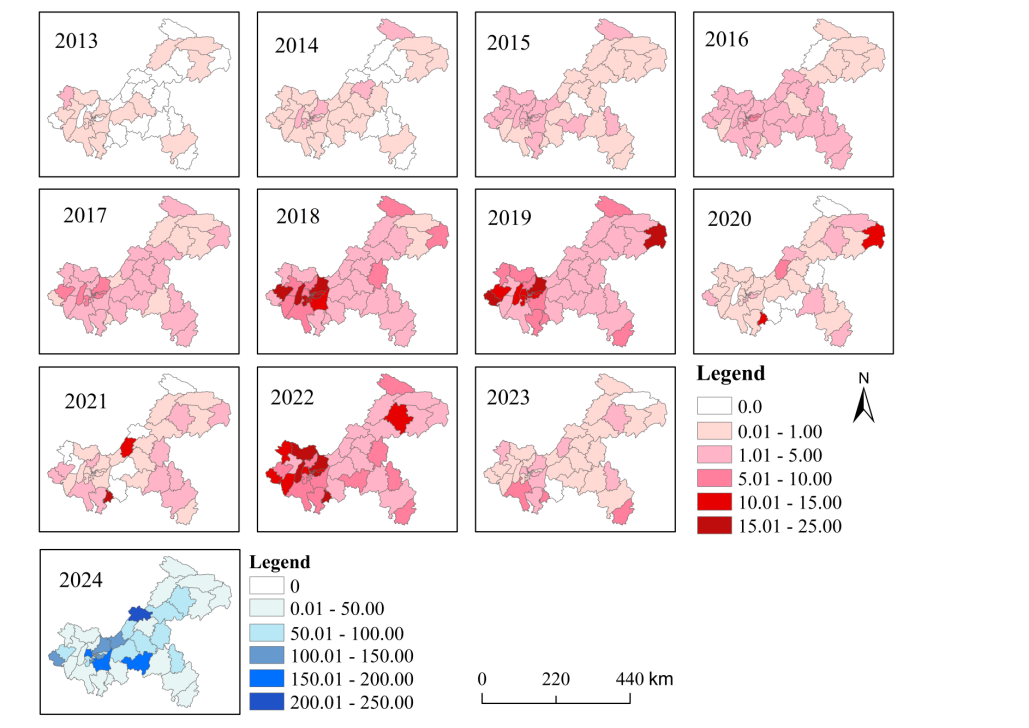


Supplementary Figure 1. District-level distribution of pertussis notification rate in Chongqing, China, 2013–2024


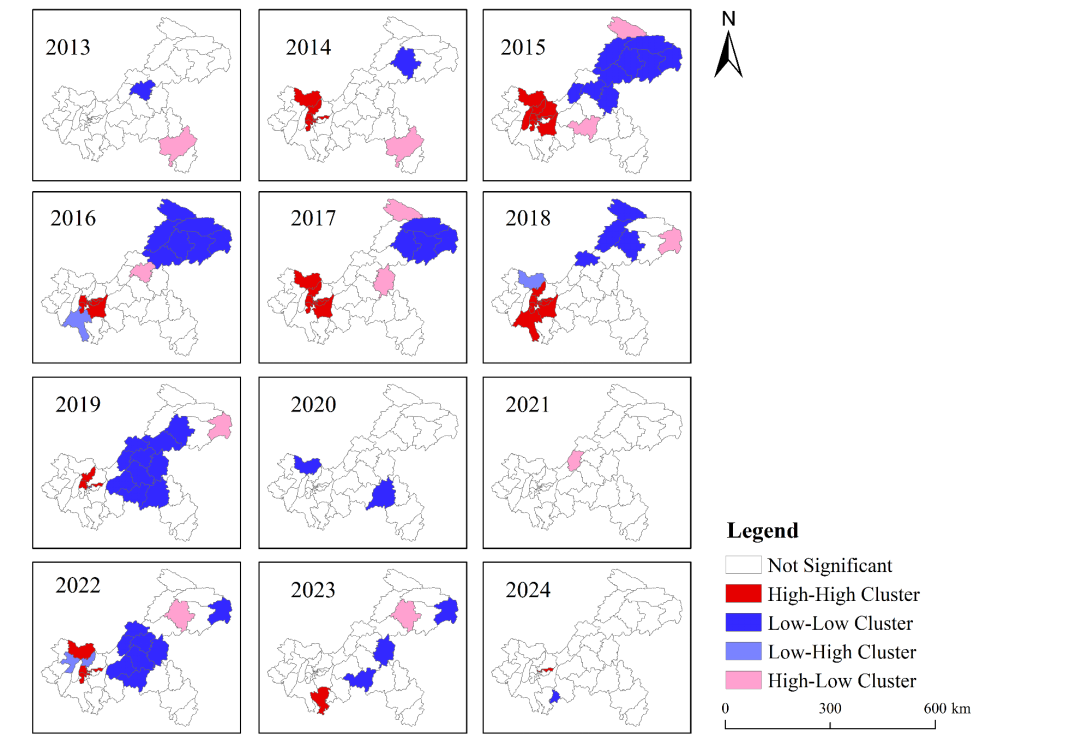


Supplementary Figure 2. Local spatial autocorrelation of pertussis notification rate in Chongqing, China, 2013–2024


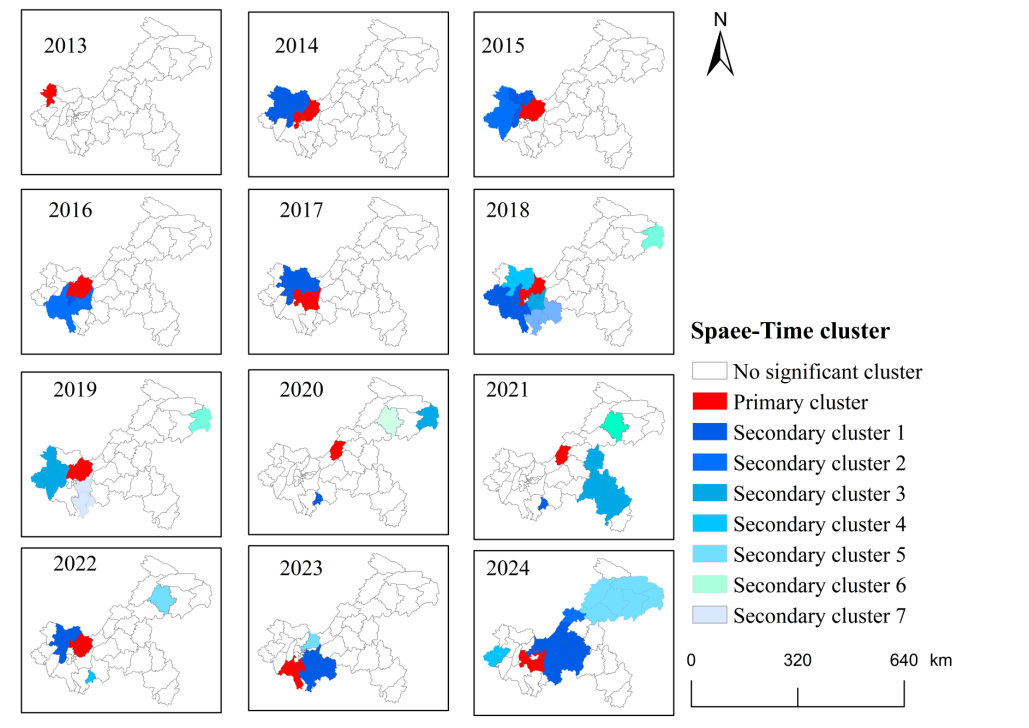


Supplementary Figure 3. Significant spatiotemporal clusters of pertussis notification rates in Chongqing, China, 2013–2024

Supplementary Table 1. Age-stratified analysis of sex differences in pertussis notification rates (2013-2024).

| Age Group | IRR | 95% CI | P-value |
| --- | --- | --- | --- |
| <1y | 1.112 | 1.064-1.163 | < 0.001 |
| 1-2y | 1.003 | 0.918-1.096 | 0.946 |
| 2-3y | 1.004 | 0.887-1.137 | 0.953 |
| 3-5y | 0.884 | 0.821-0.951 | 0.001 |
| 5-10y | 0.952 | 0.922-0.982 | 0.002 |
| 10-18y | 0.942 | 0.872-1.018 | 0.130 |
| ≥18y | 0.640 | 0.574-0.713 | < 0.001 |
| Overall | 1.040 | 1.020-1.060 | < 0.001 |

Note: IRR, incidence rate ratio. All estimates represent the rate in males compared to females (reference group)

Supplementary Table 2. Global spatial autocorrelation of annual pertussis notification rates by year in Chongqing, China, 2013–2024

| **Year** | **Moran’s I** | ***Z-score*** | ***P-value*** |
| --- | --- | --- | --- |
| 2013 | -0.02 | 0.16 | 0.370 |
| 2014 | 0.02 | 2.42 | 0.013 |
| 2015 | 0.57 | 5.52 | 0.001 |
| 2016 | 0.54 | 5.33 | 0.001 |
| 2017 | 0.35 | 3.37 | 0.002 |
| 2018 | 0.40 | 3.95 | 0.001 |
| 2019 | 0.20 | 2.22 | 0.021 |
| 2020 | -0.06 | -0.35 | 0.468 |
| 2021 | -0.10 | -0.88 | 0.096 |
| 2022 | 0.23 | 2.38 | 0.011 |
| 2023 | -0.04 | -0.19 | 0.489 |
| 2024 | 0.10 | 1.29 | 0.105 |

Supplementary Table 3. Annual spatiotemporal clusters of pertussis notification rate identified by space-time scan statistics in Chongqing, China, 2013–2024

| **Cluster type** | **Cluster center** | **Number of Clustering areas** | **Time frame** | ***LLR*** | ***RR(95%CI)*** | ***P_value*** |
| --- | --- | --- | --- | --- | --- | --- |
| The most likely cluster | Tongnan | 1 | 2013/7/1 to 2013/8/31 | 25.23 | 47.66(0.78, 2907.27) | <0.001 |
| The most likely cluster | Jiangbei | 6 | 2014/7/1 to 2014/8/31 | 20.30 | 5.37(2.44, 11.82) | <0.001 |
| Secondary cluster 1 | Tongnan | 6 | 2014/7/1 to 2014/8/31 | 9.40 | 3.89(1.66, 9.15) | 0.018 |
| The most likely cluster | Yubei | 6 | 2015/6/1 to 2015/7/31 | 28.28 | 4.32(2.50, 7.45) | <0.001 |
| Secondary cluster 1 | Beibei | 5 | 2015/3/1 to 2015/4/30 | 13.61 | 3.19(1.81, 5.63) | <0.001 |
| Secondary cluster 2 | Dazu | 7 | 2015/6/1 to 2015/7/31 | 8.98 | 2.53(1.51, 4.24) | 0.015 |
| The most likely cluster | Yubei | 6 | 2016/6/1 to 2016/7/31 | 46.92 | 4.19(2.78, 6.31) | <0.001 |
| Secondary cluster 1 | Yuzhong | 6 | 2016/2/1 to 2016/3/31 | 20.28 | 3.04(1.96, 4.71) | <0.001 |
| Secondary cluster 2 | Jiangjin | 6 | 2016/7/1 to 2016/8/31 | 9.35 | 2.18(1.45, 3.27) | 0.018 |
| The most likely cluster | Yuzhong | 7 | 2017/6/1 to 2017/7/31 | 76.58 | 4.02(2.96, 5.45) | <0.001 |
| Secondary cluster 1 | Hechuan | 5 | 2017/6/1 to 2017/7/31 | 37.03 | 3.10(2.23, 4.33) | <0.001 |
| Secondary cluster 2 | Jiangbei | 5 | 2017/3/1 to 2017/4/30 | 18.48 | 2.35(1.70, 3.24) | <0.001 |
| Secondary cluster 3 | Beibei | 5 | 2017/9/1 to 2017/10/31 | 12.06 | 2.06(1.49, 2.86) | 0.001 |
| The most likely cluster | Jiangbei | 5 | 2018/6/1 to 2018/7/31 | 135.23 | 3.87(3.10, 4.83) | <0.001 |
| Secondary cluster 1 | Rongchang | 5 | 2018/7/1 to 2018/7/31 | 89.20 | 4.56(3.25, 6.41) | <0.001 |
| Secondary cluster 2 | Nanan | 5 | 2018/3/1 to 2018/4/30 | 85.76 | 3.35(2.64, 4.26) | <0.001 |
| Secondary cluster 3 | Tongliang | 5 | 2018/4/1 to 2018/5/31 | 36.14 | 2.44(1.91, 3.12) | <0.001 |
| Secondary cluster 4 | Nanchuan | 5 | 2018/7/1 to 2018/8/31 | 22.25 | 2.20(1.68, 2.88) | <0.001 |
| Secondary cluster 5 | Wushan | 1 | 2018/8/1 to 2018/9/30 | 10.60 | 3.61(1.65, 7.88) | 0.005 |
| The most likely cluster | Yubei | 6 | 2019/5/1 to 2019/6/30 | 108.93 | 3.43(2.77, 4.26) | <0.001 |
| Secondary cluster 1 | Yubei | 6 | 2019/8/1 to 2019/8/31 | 42.12 | 2.98(2.20, 4.03) | <0.001 |
| Secondary cluster 2 | Dazu | 6 | 2019/6/1 to 2019/7/31 | 34.93 | 2.38(1.87, 3.03) | <0.001 |
| Secondary cluster 3 | Dazu | 2 | 2019/3/1 to 2019/4/30 | 23.36 | 3.10(2.01, 4.79) | <0.001 |
| Secondary cluster 4 | Yubei | 2 | 2019/2/1 to 2019/3/31 | 16.52 | 2.30(1.64, 3.22) | <0.001 |
| Secondary cluster 5 | Wushan | 1 | 2019/5/1 to 2019/6/30 | 15.46 | 4.38(1.96, 9.81) | <0.001 |
| Secondary cluster 6 | Wushan | 1 | 2019/8/1 to 2019/9/30 | 15.46 | 4.38(1.96, 9.81) | <0.001 |
| Secondary cluster 7 | Qijiang | 3 | 2019/7/1 to 2019/7/31 | 14.54 | 2.89(1.75, 4.78) | <0.001 |
| Secondary cluster 8 | Wushan | 1 | 2019/3/1 to 2019/3/31 | 10.66 | 5.08(1.63, 15.86) | 0.007 |
| The most likely cluster | Dianjiang | 1 | 2020/11/1 to 2020/12/31 | 69.38 | 27.79(4.77, 162.01) | <0.001 |
| Secondary cluster 1 | Wansheng | 1 | 2020/9/1 to 2020/10/31 | 41.75 | 36.89(2.11, 644.65) | <0.001 |
| Secondary cluster 2 | Wushan | 1 | 2020/11/1 to 2020/12/31 | 37.54 | 24.37(2.60, 228.53) | <0.001 |
| Secondary cluster 3 | Wushan | 1 | 2020/1/1 to 2020/2/29 | 17.12 | 14.23(1.53, 132.35) | <0.001 |
| Secondary cluster 4 | Wansheng | 1 | 2020/4/1 to 2020/5/31 | 15.45 | 17.95(1.06, 302.86) | <0.001 |
| Secondary cluster 5 | Wansheng | 1 | 2020/1/1 to 2020/1/31 | 13.80 | 26.34(0.48, 1430.86) | <0.001 |
| Secondary cluster 6 | Yunyang | 1 | 2020/3/1 to 2020/4/30 | 12.36 | 7.41(1.66, 33.12) | 0.001 |
| Secondary cluster 7 | Wushan | 1 | 2020/6/1 to 2020/7/31 | 11.91 | 11.13(1.24, 99.91) | 0.002 |
| Secondary cluster 8 | Wushan | 1 | 2020/9/1 to 2020/9/30 | 8.52 | 14.02(0.60, 328.23) | 0.044 |
| The most likely cluster | Dianjiang | 1 | 2021/1/1 to 2021/2/28 | 125.82 | 41.02(7.5, 224.34) | <0.001 |
| Secondary cluster 1 | Wansheng | 1 | 2021/11/1 to 2021/12/31 | 51.59 | 41.05(2.55, 660.41) | <0.001 |
| Secondary cluster 2 | Qianjiang | 4 | 2021/12/1 to 2021/12/31 | 39.62 | 12.59(3.43, 46.25) | <0.001 |
| Secondary cluster 3 | Dianjiang | 1 | 2021/4/1 to 2021/4/30 | 29.67 | 21.99(2.07, 233.34) | <0.001 |
| Secondary cluster 4 | Yunyang | 1 | 2021/12/1 to 2021/12/31 | 14.38 | 10.53(1.56, 70.83) | <0.001 |
| Secondary cluster 5 | Wansheng | 1 | 2021/6/1 to 2021/7/31 | 9.61 | 12.53(0.83, 189.06) | 0.010 |
| Secondary cluster 6 | Pengshui | 1 | 2021/8/1 to 2021/8/31 | 8.87 | 10.96(0.88, 137.13) | 0.026 |
| The most likely cluster | Yubei | 5 | 2022/5/1 to 2022/6/30 | 457.28 | 6.58(5.39, 8.04) | <0.001 |
| Secondary cluster 1 | Tongliang | 3 | 2022/5/1 to 2022/6/30 | 203.71 | 6.00(4.48, 8.05) | <0.001 |
| Secondary cluster 2 | Tongliang | 3 | 2022/2/1 to 2022/3/31 | 27.31 | 2.46(1.84, 3.28) | <0.001 |
| Secondary cluster 3 | Wansheng | 1 | 2022/2/1 to 2022/3/31 | 23.00 | 6.67(2.42, 18.41) | <0.001 |
| Secondary cluster 4 | Yunyang | 1 | 2022/7/1 to 2022/8/31 | 18.28 | 3.08(1.89, 5.01) | <0.001 |
| Secondary cluster 5 | Yunyang | 1 | 2022/4/1 to 2022/5/31 | 12.35 | 2.66(1.63, 4.35) | 0.001 |
| The most likely cluster | Jiangjin | 1 | 2023/11/1 to 2023/12/31 | 52.71 | 12.23(4.12, 36.34) | <0.001 |
| Secondary cluster 1 | Qijiang | 6 | 2023/3/1 to 2023/4/30 | 29.77 | 4.24(2.51, 7.17) | <0.001 |
| Secondary cluster 2 | Wansheng | 1 | 2023/8/1 to 2023/9/30 | 17.19 | 17.50(1.26, 243.77) | <0.001 |
| Secondary cluster 3 | Jiangjin | 1 | 2023/6/1 to 2023/7/31 | 13.99 | 5.50(1.91, 15.83) | <0.001 |
| Secondary cluster 4 | Yubei | 1 | 2023/11/1 to 2023/12/31 | 8.99 | 3.50(1.57, 7.83) | 0.022 |
| The most likely cluster | Dadukou | 6 | 2024/4/1 to 2024/5/31 | 2644.32 | 5.18(4.85, 5.54) | <0.001 |
| Secondary cluster 1 | Fuling | 7 | 2024/5/1 to 2024/6/30 | 1871.00 | 4.33(4.05, 4.63) | <0.001 |
| Secondary cluster 2 | Liangping | 1 | 2024/4/1 to 2024/5/31 | 1870.98 | 14.94(12.00, 18.61) | <0.001 |
| Secondary cluster 3 | Dazu | 2 | 2024/5/1 to 2024/6/30 | 357.55 | 3.67(3.20, 4.20) | <0.001 |
| Secondary cluster 4 | Fengjie | 6 | 2024/5/1 to 2024/6/30 | 318.00 | 2.22(2.07, 2.38) | <0.001 |
| Secondary cluster 5 | Dadukou | 6 | 2024/7/1 to 2024/7/31 | 231.10 | 2.33(2.13, 2.56) | <0.001 |

Note: LLR,Log likelihood ratio; RR,Relative risk
